# Supplementary figures and images for: Deep Metabolic Profiling Assessment of Tissue Extraction Protocols for Three Model Organisms
Source: Front Chem. 2022 Apr 25;10:869732. doi: 10.3389/fchem.2022.869732 (PMC9083328; doi:10.3389/fchem.2022.869732)

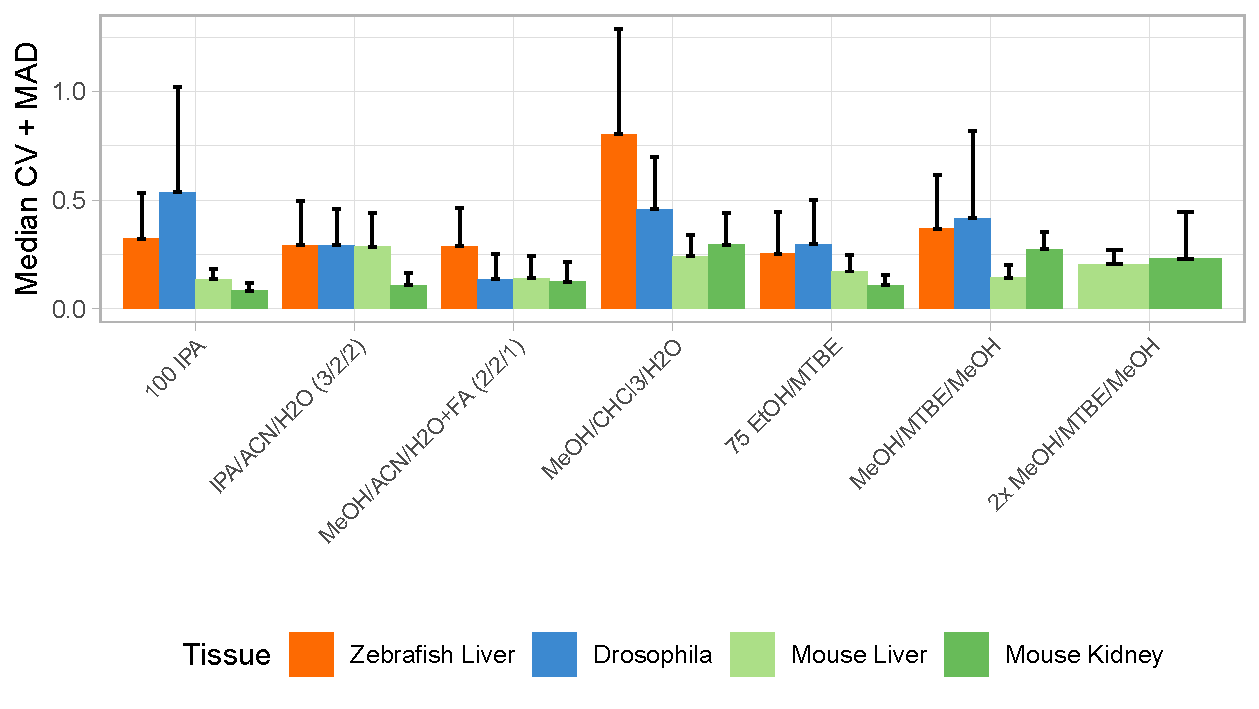

Supplement: Supplementary file 1 [file Image3.TIF]

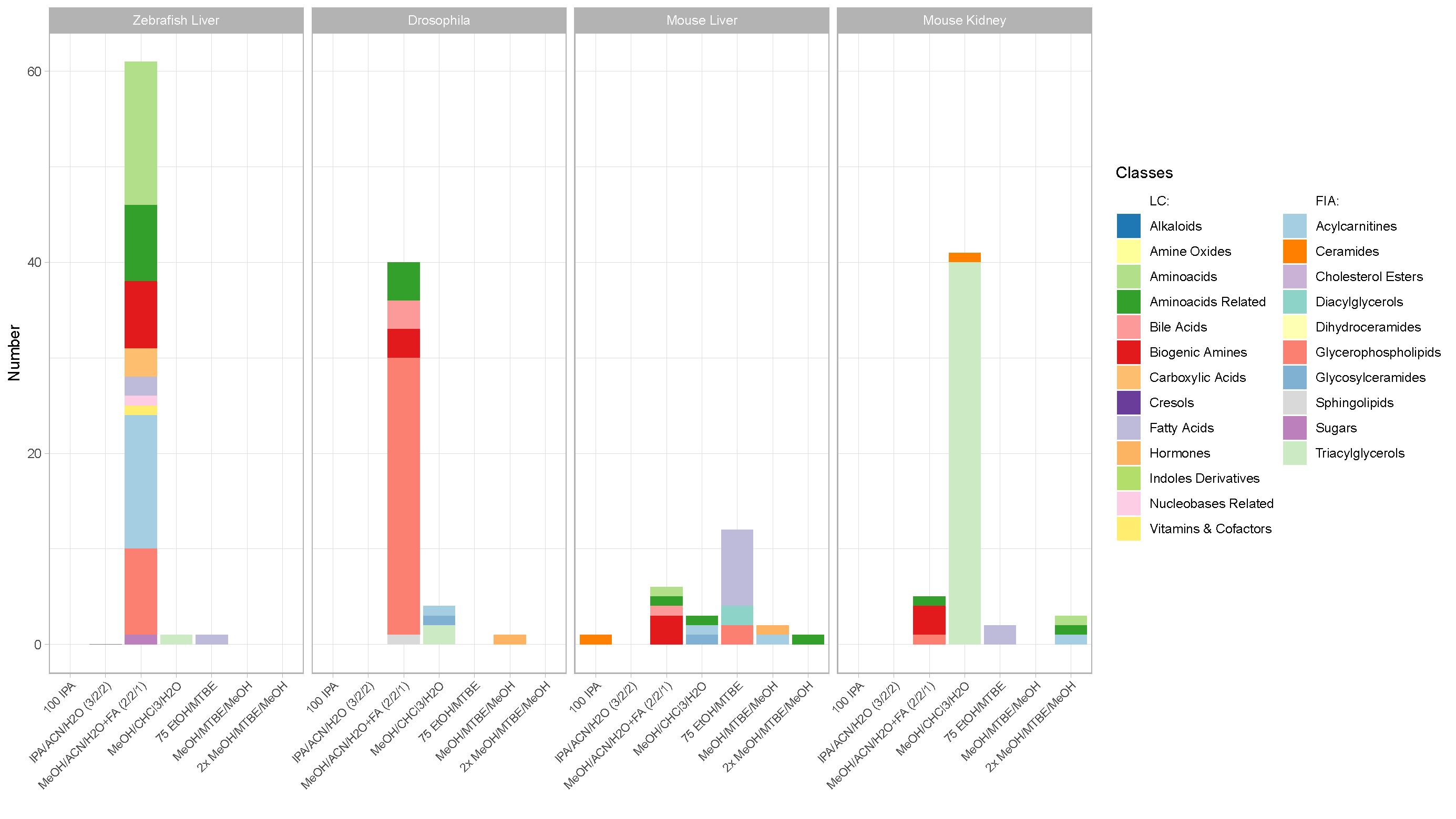

Supplement: Supplementary file 2 [file Image4.TIF]

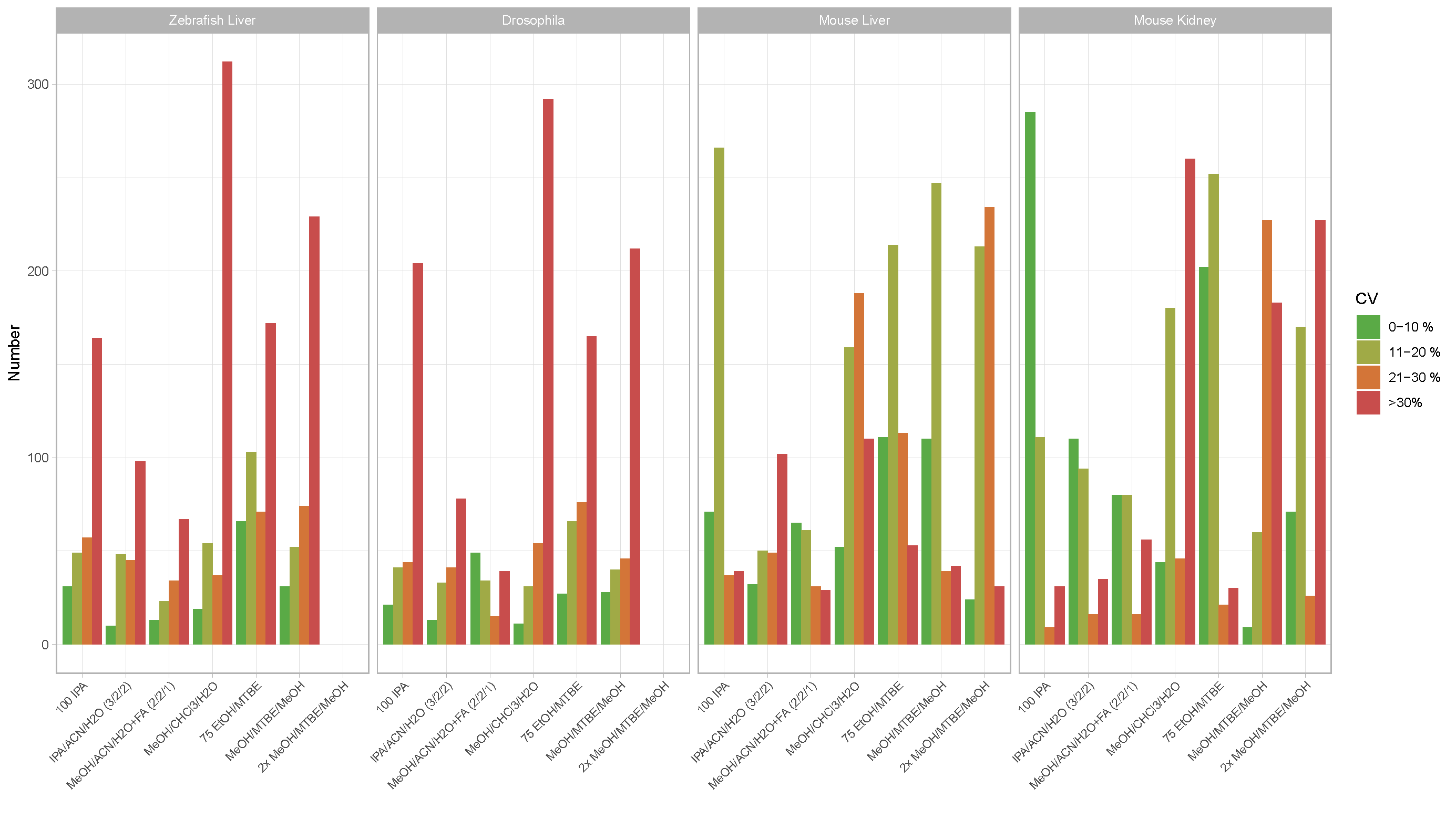

Supplement: Supplementary file 3 [file Image2.TIF]

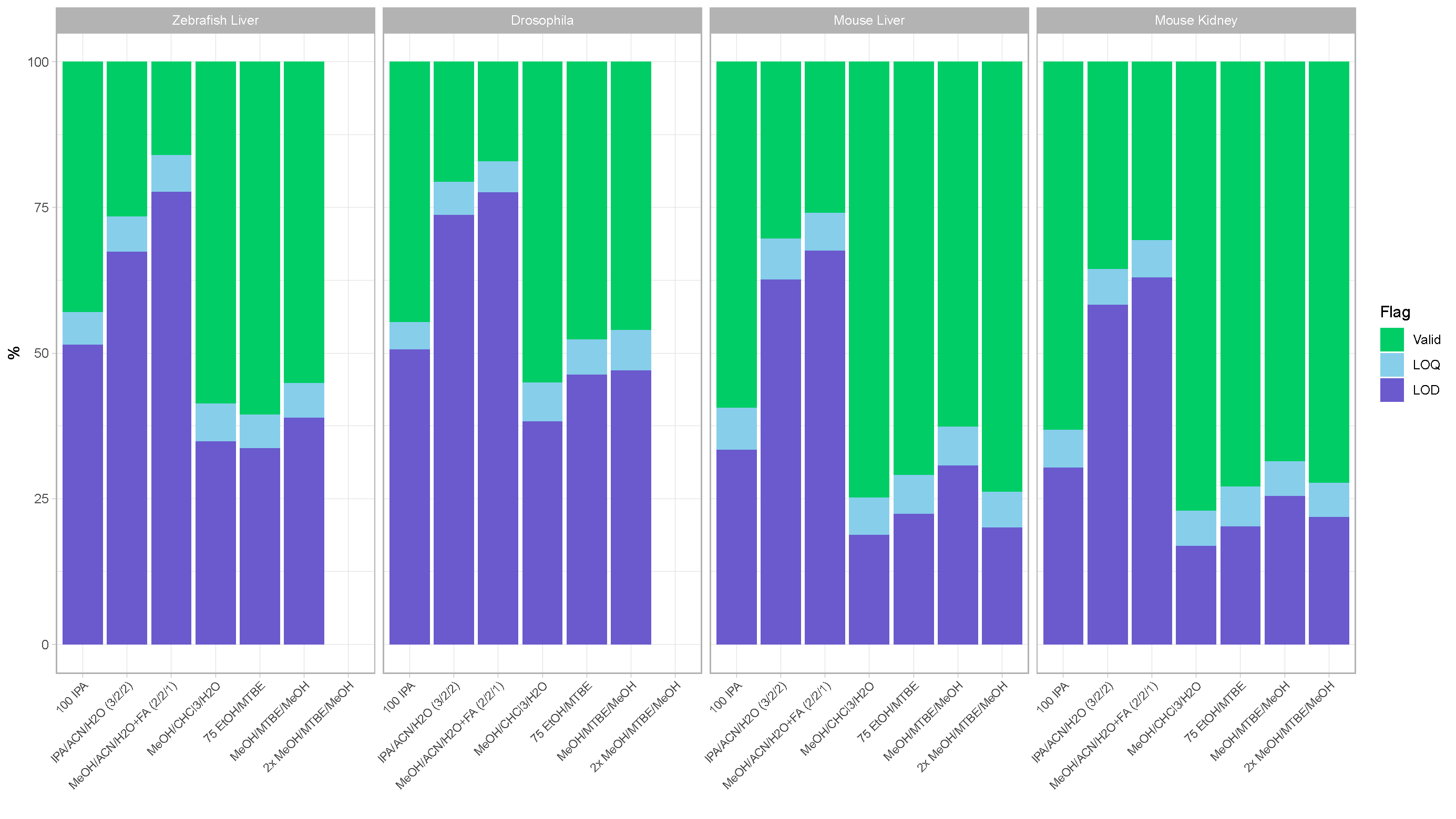

Supplement: Supplementary file 4 [file Image1.TIF]

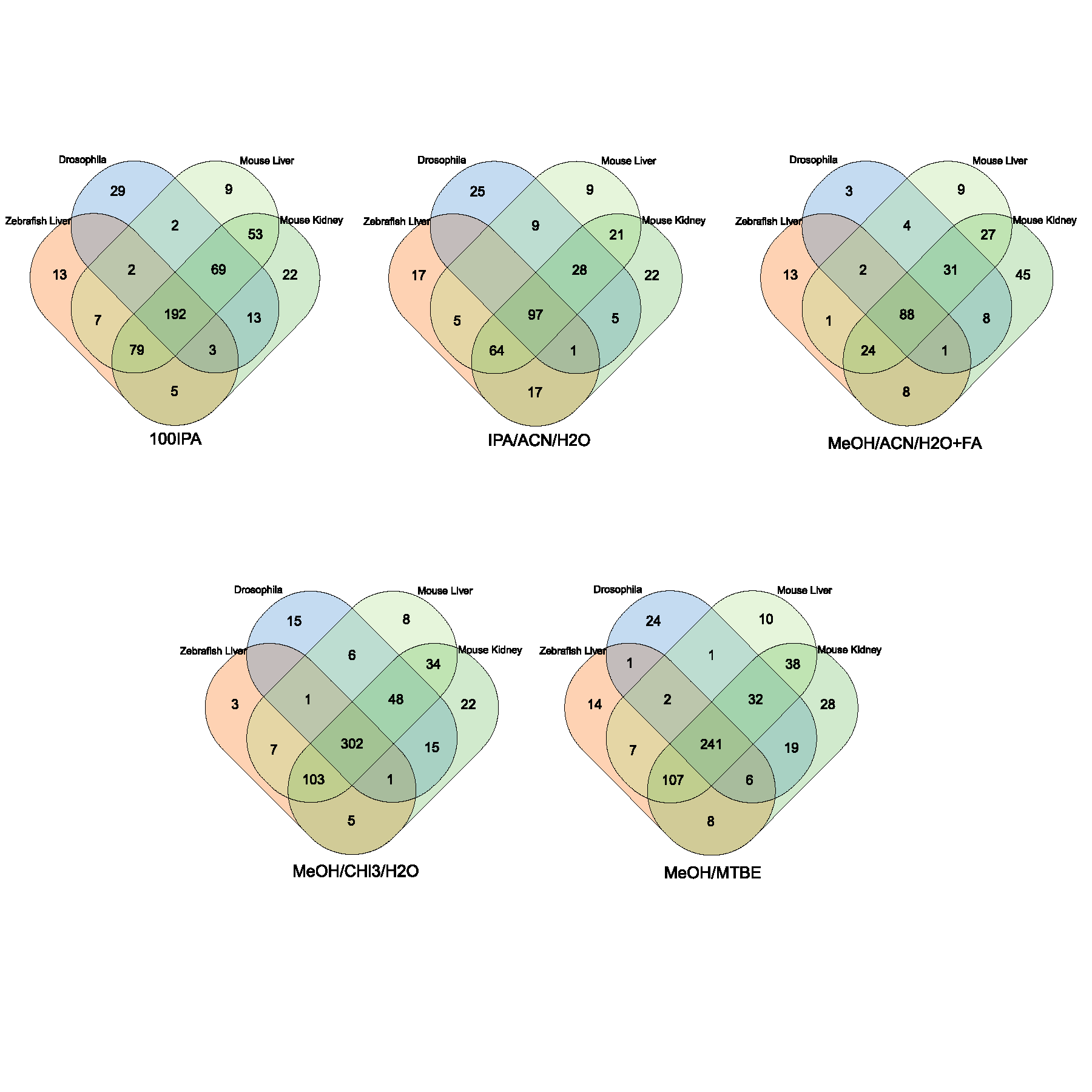

Supplement: Supplementary file 6 [file Image5.TIF]
