# Supplementary material for: Deep Metabolic Profiling Assessment of Tissue Extraction Protocols for Three Model Organisms
Source: Front Chem. 2022 Apr 25;10:869732. doi: 10.3389/fchem.2022.869732 (PMC9083328; doi:10.3389/fchem.2022.869732)
Supplement: Supplementary file 7 [file DataSheet1.docx]

# Supplementary figures


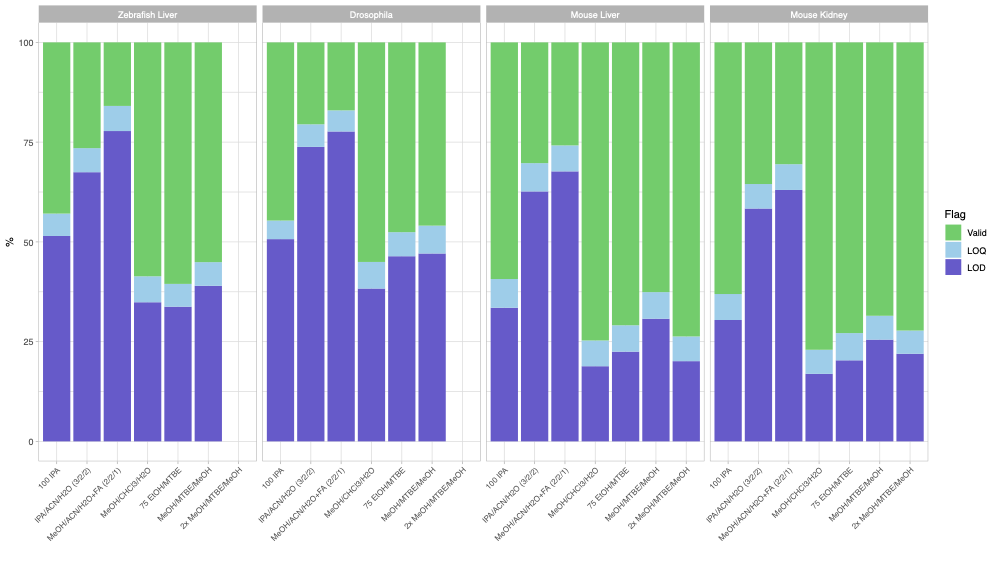


**Figure S1. Percentage of metabolite quantitation status across the seven extraction protocols per model organism and sample type.** The color indicates the status of the measurement. In dark blue the limit of detection (LOD), light blue the lower/upper limit of quantification (LOQ) and in green in the optimal quantitation range (valid). For the analyses, we considered a metabolite as detectable when at least 2 out of 3 replicates within a tested protocol were above LOD.


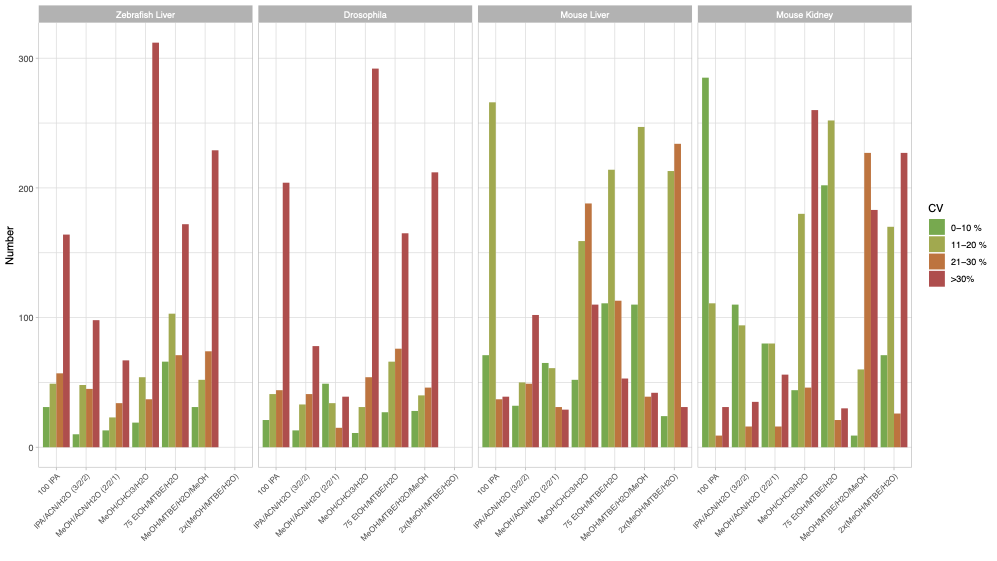


**Figure S2. Variability of metabolites across the seven extraction protocols per model organism and sample type.** Depicted is the CV% in different ranges and further rated using a color scheme from green (0-10%) to red (>30%). The number of metabolites accumulates to the total number of detectable metabolites in a given protocol.


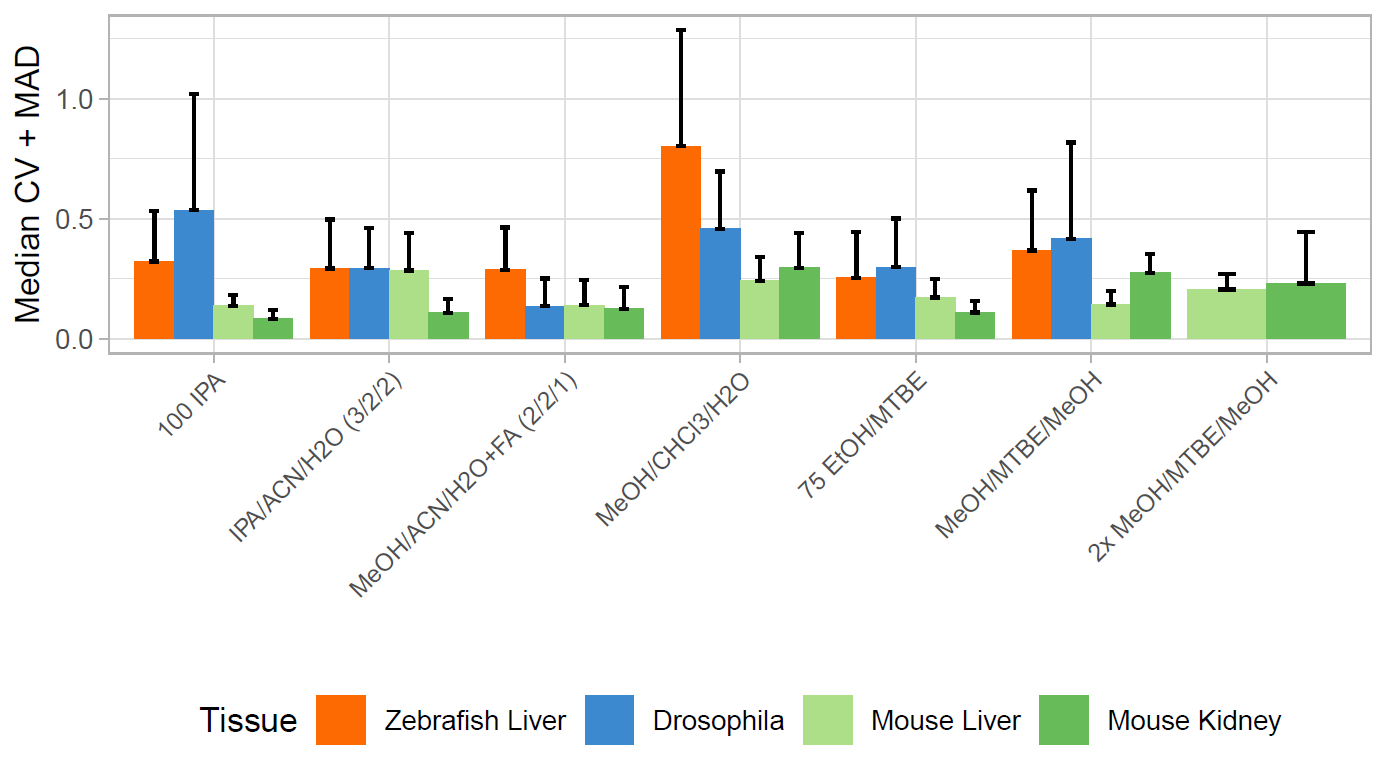


**Figure S3. Median and median absolute deviation (MAD) of the coefficient of variation (CV) across the seven extraction protocols.** Each bar represents a sample type across the different model organisms investigated. The order of extraction protocols follows Figure 1.


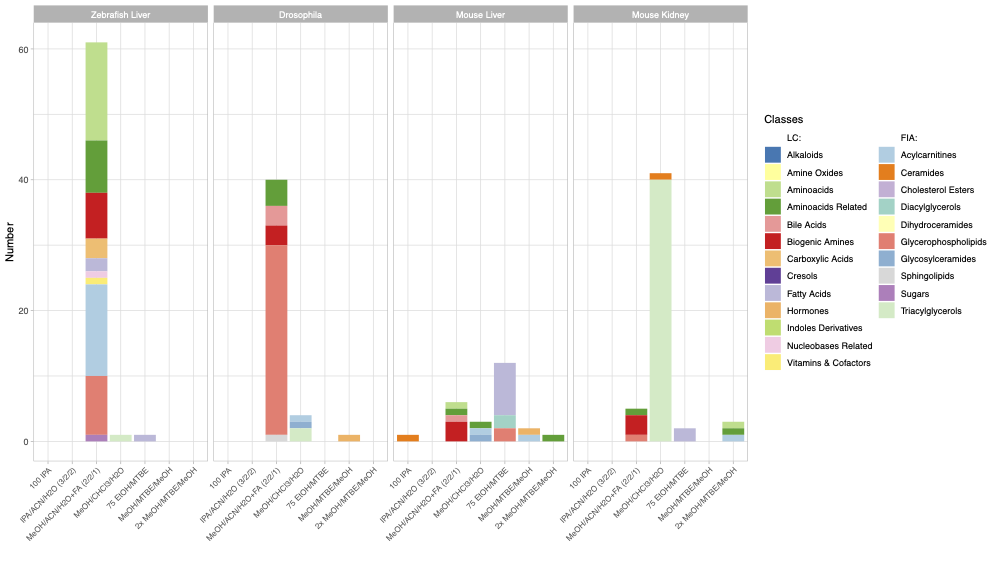


**Figure S4. Extraction protocols with exclusively high metabolite concentration across the seven extraction protocols per model organism and sample type.** Indicated by color are the different metabolite classes. The legend is categorized between the LC-MS/MS and FIA-MS/MS measurements.
